# HEK‐293 cells expressing the cystic fibrosis transmembrane conductance regulator (CFTR): a model for studying regulation of Cl^−^ transport

**DOI:** 10.14814/phy2.12158

**Published:** 2014-09-28

**Authors:** Jada C. Domingue, Mei Ao, Jayashree Sarathy, Alvin George, Waddah A. Alrefai, Deborah J. Nelson, Mrinalini C. Rao

**Affiliations:** 1 Department of Physiology and Biophysics, University of Illinois at Chicago, Chicago, Illinois; 2 Department of Biological Sciences, Benedictine University, Lisle, Illinois; 3 Department of Medicine, University of Illinois at Chicago, Chicago, Illinois; 4 Jesse Brown V.A. Medical Center, Chicago, Illinois; 5 Department of Pharmacological and Physiological Sciences, The University of Chicago, Chicago, Illinois

**Keywords:** CFTR, Cl^−^ transport, HEK‐293, TGR5

## Abstract

The Human Embryonic Kidney 293 cell line (HEK‐293) readily lends itself to genetic manipulation and is a common tool for biologists to overexpress proteins of interest and study their function and molecular regulation. Although these cells have some limitations, such as an inability to form resistive monolayers necessary for studying transepithelial ion transport, they are nevertheless valuable in studying individual epithelial ion transporters. We report the use of HEK‐293 cells to study the cystic fibrosis transmembrane conductance regulator (CFTR) Cl^−^ channel. While HEK‐293 cells endogenously express mRNA for the Cl^−^ channels, ClC‐2 and TMEM16A, they neither express CFTR mRNA nor protein. Therefore, we stably transfected HEK‐293 cells with EGFP‐CFTR (HEK‐CFTR) and demonstrated CFTR function by measuring forskolin‐stimulated iodide efflux. This efflux was inhibited by CFTR_inh_172, and the protein kinase A inhibitor H89, but not by Ca^2+^ chelation. In contrast to intestinal epithelia, forskolin stimulation does not increase surface CFTR expression and does not require intact microtubules in HEK‐CFTR. To investigate the role of an endogenous G*α*_S_‐coupled receptor, we examined the bile acid receptor, TGR5. Although HEK‐CFTR cells express TGR5, the potent TGR5 agonist lithocholic acid (LCA; 5–500 *μ*mol/L) did not activate CFTR. Furthermore, forskolin, but not LCA, increased [cAMP]_i_ in HEK‐CFTR suggesting that endogenous TGR5 may not be functionally linked to Gα_S_. However, LCA did increase [Ca^2+^]_i_ and interestingly, abolished forskolin‐stimulated iodide efflux. Thus, we propose that the stable HEK‐CFTR cell line is a useful model to study the multiple signaling pathways that regulate CFTR.

## Abstract (precis)

In this study, we characterize a HEK‐293 cell line, stably transfected with EGFP‐CFTR (HEK‐CFTR). We examined its regulation by endogenously expressed signaling pathways, in particular the cAMP and the GαS‐coupled bile acid receptor, TGR5, signaling pathways.

## Introduction

The advent of primary culture transformation to establish immortalized cell lines that can be readily studied in culture, resulted in the development of the Human Embryonic Kidney 293 (HEK‐293) cell line. In a seminal paper, Graham et al. (1977) achieved this by transfecting human embryonic kidney cells either as primary, or secondary cultures in early passages with sheared adenovirus (type 5) DNA by calcium phosphate transfection. This cell line has become the preferred tool of mammalian cell and molecular physiologists as they are of human origin and express foreign proteins readily. Amongst their advantages are the ease with which they can be cultured, transfected, and molecularly manipulated to study cloned protein function and associated signal transduction pathways. The use of HEK‐293 cells has ranged from being a mammalian cell factory for protein overexpression to being used as a tool to study structure–function correlations of a given protein. Li et al. (2007) used HEK‐293 cells to overexpress the multidrug‐resistant protein 4 (MRP4) to study spatiotemporal coupling of MRP4, PDZK1 scaffolding protein, and the cystic fibrosis transmembrane conductance regulator (CFTR). HEK‐293 cells stably transfected with the Na^+^‐dependent apical bile acid transporter (ASBT) have been used to study the roles of lipid rafts and protein trafficking in ASBT regulation (Annaba et al. 2008, 2010, 2012). In a recent study, Jensen et al. (2013) used HEK‐293 cells stably transfected with the bile acid receptor, TGR5, and transiently transfected with protease receptor 2 or *β*‐arrestins 1 and 2 to study TGR5 function and trafficking. It is important to note that the endogenously expressed HEK‐293 cell proteins may also contribute to function as well as trafficking of the overexpressed protein of interest. Therefore, there is a need to examine the function of transfected proteins against the backdrop of endogenous proteins, a concern that served as the genesis of this investigation.

A number of reductionist human cell line models have been used to study solute transport function in intestinal epithelial cells. Three in particular, derived from human colon carcinoma cells, have been used extensively to study ion transport as they form confluent monolayers exhibiting transepithelial resistance similar to that observed in native tissues. Caco‐2 cells have been used to study absorptive properties more representative of small intestinal villar and colonic surface cells, whereas, HT‐29 and T84 cells have been used to study secretory functions more representative of crypt cells (Chang et al. 2002; Ao et al. 2013; Anbazhagan et al. 2014). HT‐29 cell cultures are generally comprised of a mixture of nonmucus secreting and mucus‐secreting colonic epithelial cells with monolayers exhibiting a “medium tight” resistance of 349 ± 32 Ω cm^2^ similar to that observed in the mouse distal colon (429 ± 86 Ω cm^2^) (Gitter et al. 2000). Our laboratory has published that T84 cells can reach a transepithelial resistance of ≥950 Ω cm^2^ (Ao et al. 2013). These cells have been extensively characterized with respect to Cl^−^ secretion occurring via the concerted actions of the endogenously expressed Na^+^‐K^+^‐2Cl^−^ cotransporter, K^+^ channels, and Cl^−^ channels. Although three Cl^−^ channels, ClC‐2 (Bali et al. 2001), TMEM16A (Mroz and Keely 2012) and CFTR (Ao et al. 2013), are present in T84 cells, the predominant apical membrane channel responsible for secretion is CFTR. However, T84 cells grow slowly in culture, taking up to 2 weeks to reach confluence and have difficulty retaining polarity and function after being subjected to routine genetic manipulations including stable transfection. Given the limitations of the foregoing epithelial cell lines, we chose to study the regulation of Cl^−^ secretion, in HEK‐293 cells transfected with an EGFP‐CFTR construct to yield a stably transfected HEK‐CFTR cell line. Although previous investigators have referenced utilizing EGFP‐CFTR transfected HEK‐293 cells as a tool to propagate the recombinant vectors, there are very few published reports on the function of EGFP‐CFTR in these cells (Granio et al. 2007; Penmatsa et al. 2010). Granio et al. (2007) used HEK‐ 293 cells to propagate the EGFP‐CFTR construct before studying its function in tracheal glandular cells and in human airway epithelial cells. Penmatsa et al. (2010) checked for functional insertion of CFTR protein in HEK‐293 cells stably expressing Flag‐wt CFTR, using the iodide efflux assay.

In this study, we characterize a HEK‐293 cell line, stably transfected with EGFP‐CFTR (HEK‐CFTR) and examine its regulation by endogenously expressed signaling pathways, in particular the cAMP and the G*α*_s_‐coupled bile acid receptor, TGR5, signaling pathways (Keitel et al. 2009). TGR5 has been shown to be functionally linked to CFTR in gall bladder epithelial cells. Treatment of these cells with a synthetic TGR5 agonist led to a rise in MQAE fluorescence, indicative of an increase in Cl^−^ secretion. This increase in fluorescence was absent in TGR5 knockout mice as well as in the presence of CFTR_inh_172 (Keitel et al. 2009). Using iodide efflux as a measure of Cl^−^ transport, we report that HEK‐CFTR, but not untransfected HEK‐293 cells respond to forskolin with increased Cl^−^ transport. This stimulation is attenuated by inhibitors of CFTR and protein kinase A, but not by Ca^2+^ chelation. Forskolin stimulation does not appear to involve an increase in CFTR surface expression nor does it require intact microtubules. Bile acids have recently been recognized as having hormone‐like functions. In a variety of cell types, including enteroendocrine, neuronal, and immune cells, bile acids activate TGR5 to increase cAMP and stimulate function, such as the release of glucagon‐like peptide (Thomas et al. 2009), serotonin (Alemi et al. 2013) and inhibition of the production of pro‐inflammatory cytokines (Yoneno et al. 2013) from the above cell types, respectively. The HEK‐CFTR cells express endogenous membrane bile acid receptor, TGR5, transcript, and protein. Surprisingly they neither produce cAMP nor show an increase in Cl^−^ transport in response to the known TGR5 stimulator, lithocholic acid (LCA). However, LCA increases [Ca^2+^]_i,_ and inhibits forskolin‐stimulated Cl^−^ transport. We postulate that HEK‐CFTR cells are a useful model to study cross talk of signaling pathways regulating Cl^−^ secretion.

## Materials

Minimum Essential Media (MEM) and phenol‐free Dulbecco's Modified Eagle Medium (DMEM), fetal bovine serum (FBS), DH5alpha competent *E. coli* and geneticin were obtained from Invitrogen (Carlsbad, CA). X‐tremeGENE 9 DNA Transfection Reagent was from Roche (San Francisco, CA). Poly‐L‐lysine, lithocholic acid (LCA), H89, CFTR_inh_172, nocodazole, forskolin, carbachol, and MK571 were purchased from Sigma‐Aldrich Corp. (St. Louis, MO). HitHunter cAMP HS+ Assay was purchased from DiscoveRx (Fremont, CA).

### Antibodies

Monoclonal mouse‐anti‐human CFTR COOH‐terminus (CFTR‐C) was purchased from R&D Systems (Minnneapolis, MN). Polyclonal goat‐anti‐EGFP and rabbit‐anti‐TGR5 were from Abcam (Cambridge, MA). Monoclonal mouse‐anti‐*α*‐tubulin was purchased from Sigma‐Aldrich Corp (St. Louis, MO). Polyclonal goat‐anti‐MRP4 and mouse monoclonal GAPDH were purchased from Novus Biologicals (Littleton, CO). HRP conjugated goat‐anti‐rabbit, goat‐anti‐mouse, and bovine anti‐goat secondary antibodies were purchased from Santa Cruz Biotechnology (Santa Cruz, CA). For immunofluorescence, *SlowFade* Gold performance DAPI was purchased from Invitrogen (Carlsbad, CA). Wheat Germ Agglutinin, Alexa Fluor 594 conjugate, NucBlue Live Ready Probes Reagent were from Life Technologies (Grand Island, NY).

## Methods

### Cell culture

Human embryonic kidney (HEK)‐293 cells were grown in MEM supplemented with 10% FBS, 1% penicillin/streptomycin (100 iU/mL; 100 *μ*g/mL). The cells were incubated in a humidified atmosphere of 5% CO_2_ at 37°C. Cultures of transfected cells were stabilized in the presence of geneticin (G418, see below). T84 human colonic carcinoma cells, used as controls for RT‐PCR and immunoblot studies, were prepared as described by Ao et al. (2013).

### Transfection experiments

A hCFTR/pEGFP‐C1 plasmid consisting of wild‐type human CFTR cDNA subcloned into the multiple cloning site of the pEGFP‐C1 vector (Clonetech, Mountain View, CA), resulted in EGFP plus a 2 amino acid linker fused to the N‐terminus of hCFTR. This construct was originally generated in the laboratory of Dr. Kevin Foskett (University of Pennsylvania) and procured by Dr. D. Nelson through their collaborative studies. The construct was sequenced and verified prior to transfection. The construct was amplified by transforming DH5alpha competent *E. coli*. For transfection studies, HEK‐293 cells were seeded into 6‐well plates in the presence of the hCFTR vector using X‐tremeGENE 9 DNA Transfection Reagent. A total of 1 *μ*g DNA/well and 3 *μ*L of X‐tremeGENE 9 reagent/well were used for each transfection in antibiotic‐free media. After 48 h, cells were incubated with a medium containing 0.8 mg/mL G418 (geneticin). Resistant clones of cells were trypsinized, pooled, and maintained in a medium containing the same concentration of G418 and designated as HEK‐CFTR cells.

### Iodide effluxes

Iodide efflux studies were performed as previously described by us (Boonkaewwan et al. 2008; Anantamongkol et al. 2012; Ao et al. 2013) and are based on the Venglarik et al. method (1990) and modifications described by Chappe et al. (2003). HEK‐CFTR and HEK‐293 cells were grown in 6‐well plates coated with Poly‐L‐lysine. One million cells were seeded per well, and grown for 3 to 5 days for the cells to reach 90% confluence, at which time they were incubated with iodide‐loading buffer (containing in mmol/L: 136 NaI, 3 KNO_3_, 2 Ca(NO_3_)_2,_ 11 glucose and 20 HEPES, pH 7.4) for 1 h at room temperature (RT) in the dark. The cells were then rinsed three times with iodide‐free efflux buffer (same as the iodide loading buffer except NaNO_3_ replaced NaI). Individual wells were exposed to DMSO, LCA (5–500 *μ*mol/L), or forskolin (2–50 *μ*mol/L) ± inhibitors. Pre‐incubation with inhibitors occurred during the last 30 min of iodide loading and the inhibitors were present in the efflux buffer during the remainder of the experiment.

Iodide efflux buffer (1 mL) was then added to each well; after 2 min, the buffer was removed and saved and 1 mL of fresh efflux buffer (± inhibitor) was added to each well. Each sample that was saved contained the iodide released during the 2‐min period. The iodide concentration in each sample was determined using an iodide‐sensitive electrode (Orion 96–53; Thermo Scientific, Rockford, IL) with a pH/mV meter and a calibration curve as previously described by Boonkaewwan et al. (2008). Results are depicted either as the mean rate of iodide efflux at each 2‐min interval or as a fold change in mean cumulative iodide efflux over 12 min ± SEM relative to the value at the starting point.

### Intracellular cAMP measurements

HEK‐CFTR cells were seeded in 96‐well plates at a density of 35,000 cells per well, over night, prior to initiation of the assay. PBS with or without forskolin (10 *μ*mol/L), LCA (50 *μ*mol/L), MK571 (20 *μ*mol/L), DMSO (control), or a combination of the treatments was added to the cells and incubated at 37°C. Intracellular cAMP was measured using a HitHunter cAMP HS+ Assay according to the manufacturer's instructions (DiscoveRx, Fremont, CA). This assay uses enzyme fragment complementation (EFC) technology with two fragments of *E. coli β*‐galactosidase. The amount of luminescent signal produced is proportional to the amount of cAMP from the cell lysates. Briefly, cAMP HS+‐antibody and lysis buffer were added to the standards and cells for an incubation of 1 h at RT. The ED (Enzyme Donor) Reagent was then added to the wells and incubated for 1 h at RT. Finally, the EA (enzyme acceptor) and substrate mix were added to the wells and incubated at RT overnight in the dark. The luminescence was read after the overnight incubation using a luminescent microplate reader. The cAMP concentrations were determined from the standard curve.

### Reverse transcriptase polymerase chain reaction (RT‐PCR)

RT‐PCR was performed as previously described by Ao et al. (2013). Nontransfected (HEK‐293) and transfected (HEK‐CFTR) cells were grown to confluence in 6‐well plates or 10 cm dishes and then harvested in TRIzol reagent. Total RNA was isolated and stored in RNA‐storage solution (at −80°C). RNA was treated with DNase prior to synthesis of cDNA. RNA concentrations and purity were measured by Nano‐drop UV‐Vis Spectrophotometer (Thermo Scientific) at 260 nm. One *μ*g of RNA was used for cDNA synthesis by superscript II reverse transcriptase. Primer pairs used for PCR were as follows:

CFTR (F: CAAGGAGGAACGCTCTATCG and R: GCCTTCCGAGTCAGTTTCAG; 558 bp);
CLC‐2 (F:AGTGGGAGGAGCAACTA and R:TGGGTCAGATTCCAGGTAGG; 557 bp);
TMEM16A (F:GGCTTTCCTGCTGAAGTTTG and R:CGATGTCTTTGGCTCTGACA; 505 bp);
MRP2 (F: GAGCAAGTTTGAAACGCACA and R: AGCCGCAGTGAATAAGAGGA; 397 bp); MRP3 (F: TGTGCTAGCTGATGGACAGG and R: TGTCACCTGCACCTTCTCTG; 340 bp); MRP4 (F: CCATCTGTGCCATGTTTGTC and R: CCACAATGCCAACCTTTTCT; 363 bp); and TGR5 (F: CTCAGTCCTGGCCTATGAGC and R: TAACGGCCAGAGGAGCTTTA; 399 bp).

The PCR was performed at an annealing temperature of 59°C for 36 cycles.

### Immunoblotting

Cells were grown to confluence as described above; when grown in 6‐well plates, 3 wells were pooled together as a single sample. Cells were lysed in a buffer (LB) containing in mmol/L: 25 Tris‐HCl (pH 7.4), 1 EDTA, 2 MgCl_2_, 5 *β*‐mercaptoethanol, 1 DTT, and 10 *μ*L/mL LB protease inhibitor cocktail. The lysates were sonicated on ice (25 sec; Branson Sonifier Cell Disruptor Model 350, Branson Ultrasonics Co., Danbury, CT) and centrifuged at 3000 × g for 10 min (4°C). The pellet containing nuclei and unlysed cells was discarded and the supernatant, total cell lysate, was further fractionated into membrane and cytosolic fractions, 30 min at 10,000 × g (4°C). The pelleted membrane fraction was resuspended in lysis buffer. Protein concentrations were measured using the Bio‐Rad Protein Assay protocol. Cell fractions (30–50 *μ*g of protein) were separated by SDS‐polyacrylamide gel (7.5% or 4–15%) electrophoresis and the proteins subjected to western blotting as described previously (Anantamongkol et al. 2012; Ao et al. 2013). Blots were blocked with 5% milk (1 h, RT) and incubated with primary antibodies (in 1% milk, O/N, 4°C). After washing with Tris‐Buffered Saline (pH 7.4) containing 0.1% Tween 20, blots were exposed to horseradish peroxidase (HRP)‐conjugated secondary antibodies (1:10,000 dilution; 1 h, RT) and visualized with Pierce SuperSignal West Pico Chemiluminescent Substrate kit (Thermo Scientific). When needed, the membranes were stripped in a buffer containing 100 mmol/L ß‐mercaptoethanol, 2% SDS, 62.5 mmol/L Tris·HCl (pH 6.7) for 30 min, 55°C, and reprobed with different primary antibodies as described above. The secondary antibody exposure and visualization of the reaction product were as described above. Immunoblot bands were quantified by Image J software (Rasband, W.S., U.S. National Institutes of Health, Bethesda, MD) after scanning densitometry. The concentrations of primary antibodies used are listed in the figure legends.

### Cell surface biotinylation

Cell surface biotinylation was performed as described by Gill et al. (2007). Briefly, confluent HEK‐CFTR cells were equilibrated in serum‐free media and treated with 10 *μ*mol/L forskolin or DMSO (0.1%) for 0–10 min at RT. Cells were placed on ice, washed with a modified PBS (PBS‐M: PBS+ 0.1 mmol/L CaCl_2_ and 1 mmol/L MgCl_2_), and exposed to biotin (1.5 mg/mL) in borate buffer (in mmol/L: 10 boric acid pH 9.0, 154 NaCl, 7.2 KCl, and 1.8 CaCl_2_,) for 1 h, 4°C, in the dark. The cells were treated with quenching buffer (PBS‐M + 100 mmol/L glycine, 20 min, 4°C) to bind any excess biotin, washed in PBS‐M and collected by centrifugation at 13,000 × g (10 min, 4°C). The cells were lysed in RIPA buffer (in mmol/L: 150 NaCl, 50 Tris‐HCl pH 7.6, 5 EDTA, 1% Triton X‐100, 0.1% SDS, protease inhibitor cocktail), sonicated (2 × 30 sec) and centrifuged at 13,000 × g (4°C, 10 min) to yield the total cell lysate. Protein concentrations were determined by Bio‐Rad Protein Assay. One mg of protein from each treatment group was added to Neutravidin beads (Thermo Scientific) that were pre‐equilibrated with RIPA buffer. Samples were incubated overnight (4°C) in an Eppendorf rotoshaker. The beads were collected by centrifugation at 13,000 × g (4°C, 10 min) and the supernatant containing the nonbiotinylated fraction was saved. The beads were washed with RIPA buffer (5000 × g, 5 min, 4°C ×3) and resuspended in 2X loading dye (Bio‐Rad, Hercules, CA). Nonbiotinylated fractions were diluted in 2X loading dye. Nonbiotinylated, and biotinylated fractions were heated (60°C, 15 min) and resolved by SDS‐PAGE, and immunoblotting as described above. The blots were probed with CFTR‐C antibody (1:1000) to assess CFTR expression. To check for nonbiotinylated protein contamination of the biotinylated fraction, blots were stripped and reprobed with GAPDH antibody (1:2000).

### Preparation of detergent‐soluble and insoluble tubulin

Detergent‐soluble and ‐insoluble tubulin was prepared according to Yu et al. (2009). To establish a protocol for destabilizing microtubules in HEK‐CFTR cells, the distribution of detergent‐soluble (monomeric) and detergent‐insoluble (microtubule) *α*‐tubulin was examined under four conditions of nocodazole exposure. HEK‐CFTR cells were grown in 10‐cm dishes and exposed to one of the following treatment regimens: I. 4°C, 30 min to destabilize microtubules followed by incubation with nocodazole on ice 30 min and then at RT, 1 h; II. Nocodazole on ice for 30 min followed by incubation at RT, 1 h; III. 4°C for 30 min followed by incubation with nocodazole at RT, 1 h; IV. Incubation with nocodazole at RT, 1 h. After these regimens, the cells were rinsed with PBS and once with extraction buffer (EB; in mmol/L: 100 PIPES pH 6.75, 1 MgSO_4_, 2 EGTA, 0.1 EDTA, and 2000 glycerol). Cells were subsequently extracted ×2, 8 min each with 750 *μ*L EB containing 0.1% Triton X‐100 and protease inhibitors and the fractions were collected to yield the detergent‐soluble fraction. The detergent‐insoluble fraction remaining on the plate was treated with lysis buffer (in mmol/L: 25 Na_2_HPO_4_ pH 7.2, 400 NaCl, and 0.5% SDS), sonicated and centrifuged for 10 min (2000 × g), and the DNA‐containing pellet was discarded. Equal amounts of detergent‐soluble and insoluble proteins from each treatment group were subjected to SDS‐PAGE and immunoblotting as described above using α‐tubulin antibody (1:2500 dilution).

### [Ca^2+^]_i_ measurements

HEK‐CFTR cells were grown in 35 mm glass‐bottom dishes (Mat‐Tek Corporation, MA) coated with poly‐L‐lysine. One million cells were seeded per dish, and [Ca^2+^]_i_ was measured after 4 days, when the cells reached 90% confluency. Briefly, the cells were loaded with 5 *μ*mol/L Fura‐2‐AM in serum‐free MEM for 1 h in a tissue culture incubator at 37°C, and washed 3 times with Krebs–Ringer‐Hepes buffer (KRH; in mmol/L: 120 NaCl, 5.4 KCl, 0.8 MgCl_2_, 1 CaCl_2_, 11.1 glucose and 20 Hepes pH 7.4). One mL of fresh KRH was added per dish, and the dish was set up on a dual channel temperature controlled (Warner Instruments, Hamden, CT) platform of a fluorescence microscope (Olympus IX51, Olympus America, Center Valley, PA). Secretagogues in KRH buffer were delivered to the cells by a perfusion and vacuum system (Warner Instruments). Ca^2+^ signals were captured using a Q‐8 spectrofluorometer system (Photon Technology International, Edison, NJ), and are reported as fluorescence ratios (Exλ: 340/380 nm; Emλ: 505 nm) (Venkatasubramanian et al. 2001; Carlos et al. 2007).

### Live cell imaging and vesicle trafficking

HEK‐CFTR cells were grown as described for Ca^2+^ measurements. The cells were washed with PBS, and incubated with 1 mL of phenol‐free DMEM containing 5 *μ*g Wheat Germ Agglutinin (WGA), Alexa Fluor 594 conjugate (Life Technologies, Grand Island, NY) for 10 min at 37°C in a tissue culture incubator. The cells were then washed five times with 1 mL of phenol‐free DMEM. One mL of phenol‐free DMEM containing 2 drops of NucBlue Live ReadyProbes Reagent (Life Technologies) was then added to the dish. Next, the dish was mounted on a Zeiss LSM 710 confocal microscope (META) (Zeiss, Oberkochen, Germany) equipped with a PeCon temperature controller (PeCon GmbH, Erbach, Germany) to keep the temperature at 37°C. After a 2‐min incubation with NucBlue, 3 images were taken as baseline using diode UV laser, (Exλ: 405 nm, Emλ: 420–480 nm); Argon laser (Exλ: 488 nm, Emλ: 500–550 nm), and DPSS 561 laser (Exλ: 561 nm, Emλ: 600–650 nm). Then DMSO or forskolin were added to the cells, and images were captured as time series using ZEISS microscope software Zen. A 63×/1.46 Oil objective was used, and the final magnification is 126 times. For CFTR vesicle tracking, z‐stack images were also acquired in addition to time series to generate 4D images. The vesicles were detected using the “spot detection” function of Imaris (Bitplane USA, South Windsor, CT) and were tracked over time. To optimize sample size, vesicles ≥0.71 *μ*m in diameter were tracked. Based on the relative size of the cells, vesicular tracking was done over a length of 1–15 *μ*m. Vesicle speeds and other parameters were exported into Excel.

### Statistical analysis

Data from at least three individual experiments were analyzed and presented as mean ± SEM. Statistical significance was determined using paired Student's t‐test, 1‐way ANOVA, or 2‐way ANOVA and values of *P *<**0.05 were considered statistically significant**.**

## Results

### Expression of CFTR in transfected and native HEK‐293 cells

**Figure 1.**
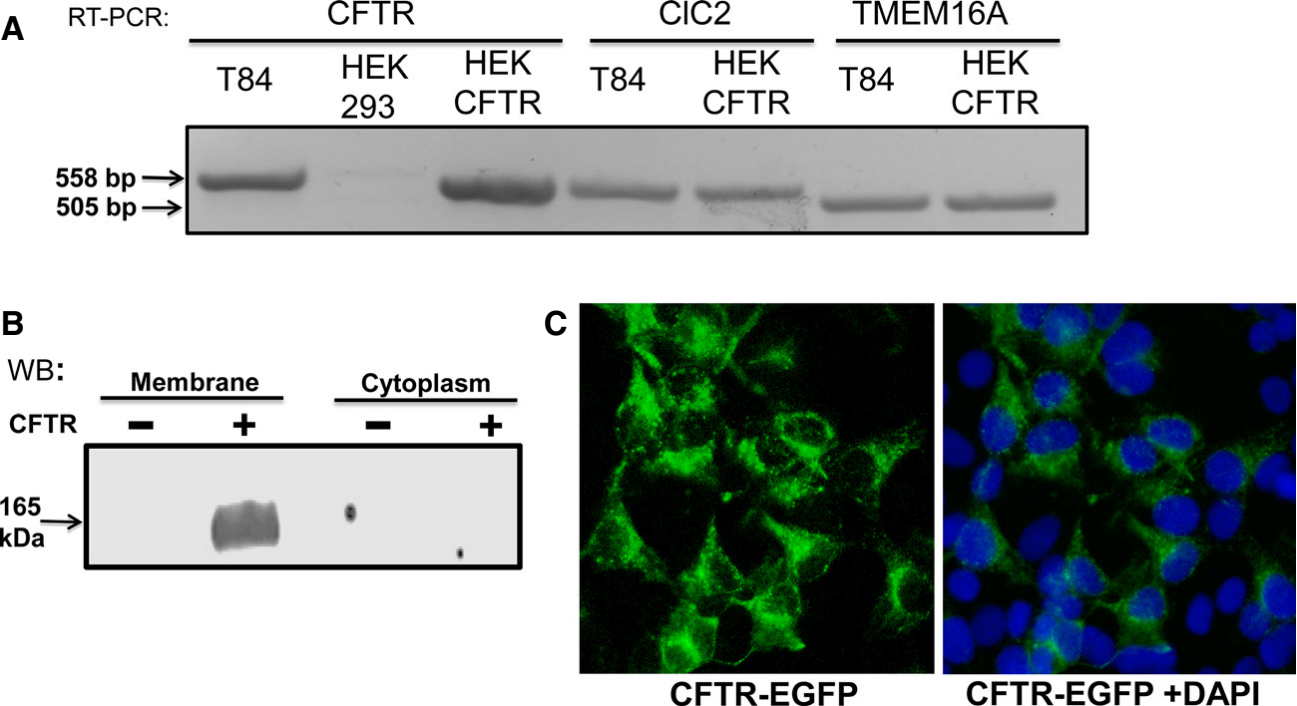
Cl^−^ channel expression in HEK‐293 and CFTR transfected HEK cells (HEK‐CFTR). (A) Detection of mRNA for transcripts of Cl^−^ channels CFTR, ClC‐2 and TMEM16A by Reverse Transcriptase (RT)‐PCR in HEK‐CFTR cells. T84 colonic epithelial cells were used as a positive control. The anticipated size of the mRNA transcripts are as follows in base pairs: CFTR: 558; ClC‐2: 557; and TMEM16A: 505. (B) Detection of CFTR protein in HEK‐293 and HEK‐CFTR cells by Western blotting (Ab: monoclonal mouse‐anti‐CFTR‐C; 1:1000 dilution; estimated size 165 kDa). (C) Representative epifluorescence image of HEK‐293 cells stably transfected with EGFP‐CFTR (HEK‐CFTR) and stained with DAPI for nuclear visualization. Images in each figure are representative of *n *≥**3 experiments.

As the focus of this study is to examine the regulation of CFTR function in a stably transfected cell line, we first determined if native HEK‐293 cells express CFTR. As shown in Fig. 1, HEK‐293 cells neither express CFTR mRNA transcript (1A) nor CFTR protein (1B). Therefore, we stably transfected these cells with human CFTR using a pEGFP‐C1 vector. Transfection resulted in robust expression of CFTR as demonstrated by mRNA transcript expression (1A), protein expression (1B), and epifluorescence microscopy (1C). DAPI was used to stain nuclei. Furthermore, the fluorescence and immunoblot data suggest that CFTR is expressed in membrane fractions of the CFTR transfected cells (Fig. 1). The EGFP‐CFTR protein was also detectable with EGFP antibody (1:1000; data not shown). These cells will be referred to as HEK‐CFTR cells. We also examined if HEK‐293 cells possess Cl^−^ channels other than CFTR and probed for ClC‐2 and TMEM16A transcripts, using T84 cells as a positive control. HEK‐CFTR (Fig. 1A) and HEK‐293 cells (data not shown) express both ClC‐2 and TMEM16A mRNA.

### Forskolin induces a dose‐dependent iodide efflux in HEK‐CFTR cells

**Figure 2.**
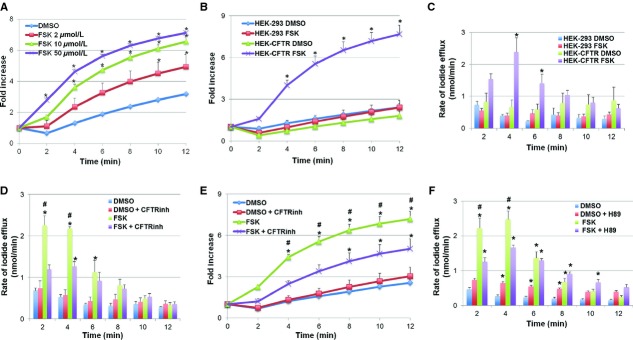
Effect of forskolin on iodide efflux in HEK‐CFTR and HEK‐293 cells. (A) Dose‐dependent effect of forskolin (FSK; 2–50 *μ*mol/L) on iodide efflux in HEK‐CFTR cells. (B) Effect of FSK (10 *μ*mol/L) on iodide efflux in HEK‐293 and HEK‐CFTR cells. Iodide efflux was measured at 2‐min intervals for 12 min and cumulative iodide efflux represented as fold increase over basal flux at time = 0 min. (C) Rate of iodide efflux (nmol/min) after addition of FSK in HEK‐293 and HEK‐CFTR cells. (D and E). Effect of CFTR_inh_172 (20 *μ*mol/L) on iodide efflux in HEK‐CFTR cells stimulated with FSK; HEK‐CFTR cells were pretreated with DMSO (0.1%) or CFTR_inh_172 for 30 min during iodide loading. Effect of CFTR_inh_172 on rate of iodide efflux shown in D and on fold increase of the mean cumulative iodide efflux over 12 min shown in E. (F) Effect of PKA inhibitor, H89 (30 μmol/L), on the rate of iodide efflux in HEK‐CFTR cells stimulated with FSK; HEK‐CFTR cells were pretreated with DMSO or H89 for 30 min during iodide loading. Data represented are mean values ± SEM relative to value at starting point of *n *≥**3 experiments. *P <**0.05 versus DMSO control; ^#^*P *<**0.05 versus treatment+inhibitor.

To determine if transfected CFTR was functional, we used the iodide efflux assay, a method originally described by Venglarik et al. (1990). Briefly, cells were loaded with iodide, then extracellular iodide was removed and the rate of iodide efflux ± putative activators or inhibitors of Cl^−^ channels is determined. This procedure has been used in a variety of epithelial and nonepithelial cells to measure changes in permeability of a Cl^−^ channel, such as CFTR, that is permeable to both Cl^−^ and I^−^ (Venglarik et al. 1990; Long and Walsh 1997; Penmatsa et al. 2010). We have effectively used this method to study Cl^−^ transport both in HC‐11, mouse mammary epithelial cells, which do not develop resistive monolayers (Anantamongkol et al. 2012) and in T84 human colonic epithelial cells, which form high resistance monolayers (Boonkaewwan et al. 2008; Ao et al. 2013). CFTR is activated primarily by cAMP, and we and others have shown that in T84 cells, 10 *μ*mol/L of the adenylate cyclase activator, forskolin, causes maximal Cl^−^ secretion (Ao et al. 2013). To establish the optimal dose of forskolin action in HEK‐293 cells, we examined the effects of 2–50 *μ*mol/L forskolin on iodide efflux in HEK‐CFTR cells. As shown in Fig. 2A, forskolin caused a dose‐dependent increase in iodide efflux as compared to DMSO as control (*n *=**4). Based on these results, we estimate the EC50 of forskolin to be approximately 3.5 *μ*mol/L in HEK‐CFTR cells. Although 50 *μ*mol/L forskolin resulted in the largest increase in iodide efflux, the rates were not statistically different than the effect of 10 *μ*mol/L. To avoid the nonspecific effects of high concentrations of forskolin (Zimmerman et al. 2012), in the remaining experiments, 10 *μ*mol/L forskolin was used to assess CFTR function.

### Forskolin does not increase iodide efflux in nontransfected HEK cells

To determine if the effects of forskolin reflected CFTR function, we compared its effects on iodide efflux in HEK‐CFTR and nontransfected HEK‐293 cells. As shown in Fig. 2B, 10 *μ*mol/L forskolin had no effect on the cumulative I^−^ efflux on HEK‐293 cells over 12 min, as compared to DMSO and in contrast to its action on HEK‐CFTR cells. The rates of iodide efflux vary over the 12 min and as shown in Fig. 2C, forskolin caused a sharp increase in iodide efflux beginning at 2 min and peaking at 4 min.

The forskolin response was significantly different from DMSO in HEK‐CFTR cells throughout the duration of the experiment (Fig. 2B). There was no difference in the cumulative iodide efflux in response to DMSO alone in HEK‐293 and HEK‐CFTR cells (Fig. 2B). These results demonstrate that the transfected CFTR is functional as measured by iodide efflux response to forskolin, and that forskolin does not alter iodide efflux in nontransfected HEK cells. Equally important, these results suggest that although HEK‐293 cells possess ClC‐2 and TMEM16A transcript, forskolin‐stimulated efflux occurs through CFTR when present.

### CFTR_inh_172 inhibits forskolin‐induced iodide efflux

To further confirm that the iodide efflux stimulated by forskolin is CFTR dependent, HEK‐293 and HEK‐CFTR cells were pretreated with CFTR_inh_172 (20 *μ*mol/L) for 30 min during iodide loading. CFTR_inh_172 was also present in the iodide efflux buffer during the experiment. In HEK‐CFTR cells, forskolin significantly increased rates of iodide efflux compared to DMSO even in the presence of CFTR_inh_172, albeit to a lower extent (Fig. 2D). CFTR_inh_172 reduced the cumulative iodide efflux (Fig. 2E) at 2 min by 47%, 44% at 4 min, and by 30% at the final time point (12 min). However and more importantly, CFTR_inh_172 inhibited the increase in the forskolin‐stimulated efflux at 2 and 4 min by 47% and 42%, respectively (Fig. 2D). In addition, the cumulative efflux response was attenuated at all time points, but not completely inhibited by CFTR_inh_172 (Fig. 2E). CFTR_inh_172 had no effect on the iodide efflux in DMSO‐treated cells. Not surprisingly, there was also no difference in iodide efflux in HEK‐293 cells ± forskolin ± CFTR_inh_172 (data not shown). For the remainder of the studies, we focused on characterizing Cl^−^ transport in HEK‐CFTR cells.

### cAMP but not Ca^2+^ signaling is necessary for forskolin‐induced iodide efflux

To confirm that forskolin, a known activator of adenylyl cyclase, increases cAMP, HEK‐CFTR cells were pretreated with forskolin for 5 min and intracellular cAMP measured as described. Forskolin caused a robust increase in cAMP in HEK‐CFTR cells (Fig. 6C).

To determine if cAMP‐dependent protein kinase (PKA) is involved in forskolin action, HEK‐CFTR cells were pretreated with the PKA inhibitor H89 (30 *μ*mol/L) for 30 min during iodide loading and continuously thereafter throughout the experiment. While H89 had no effect on iodide efflux in DMSO‐treated cells, it significantly inhibited the forskolin‐stimulated efflux at 2 and 4 min by 43% and 33%, respectively (Fig. 2F).

In contrast, the Ca^2+^‐chelator BAPTA‐AM had no effect on forskolin‐stimulated ion transport (maximal cumulative efflux at 12 min, in fold increase of start value: Forskolin [10 *μ*mol/L]: 6.2 ± 0.9, Forskolin +BAPTA [20 *μ*mol/L]: 5.9 ± 0.5). Furthermore, forskolin did not alter [Ca^2+^]_i_ in these cells (Fig. 6D*)*. These results suggest that the action of forskolin on iodide efflux is dependent on protein kinase A, but not on changes in [Ca^2+^]_i_.

### Activation of CFTR by forskolin is independent of microtubules in HEK‐CFTR cells

**Figure 3.**
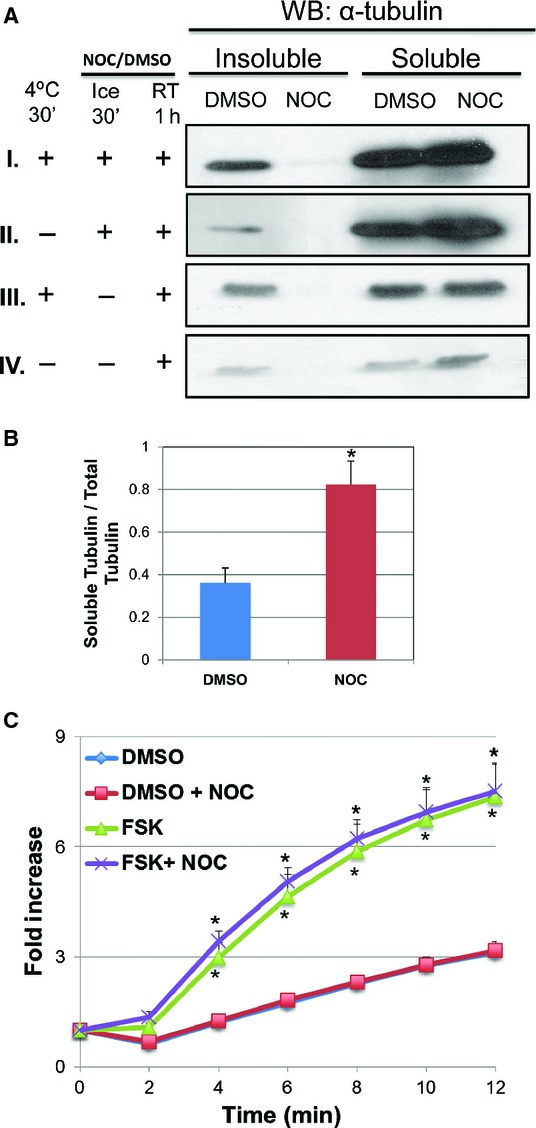
Effect of microtubule destabilization by nocodazole on forskolin‐induced iodide efflux. (A) HEK‐CFTR cells were pretreated with nocodazole (NOC; 33 *μ*mol/L) for 1 h at room temperature (RT) preceded by either incubation at 4°C for 30′, or addition of NOC for 30′ on ice, or a combination of the two. These representative blots show the 0.1% Triton X‐100 soluble and insoluble *α*‐tubulin (Ab: monoclonal mouse‐anti‐*α*‐tubulin; 1:2,500 dilution; estimated size: 50 kDa). (B) The ratio of soluble tubulin to total tubulin (insoluble + soluble tubulin) was analyzed by densitometry of the DMSO and Nocodozole signals for all four conditions. Representative values for protocol ‘I” (the top most blot: 4°C for 30′ → NOC for 30′ on ice →NOC for 1 h at RT) are shown (data not shown for “II to IV”). (C) Effect of nocodazole on iodide efflux in HEK‐CFTR cells. Cells were pretreated with NOC for 1 h at RT during iodide loading, and then stimulated with forskolin (FSK; 10 *μ*mol/L). The data represent the fold change of mean cumulative iodide efflux ± SEM relative to value at starting point. Here *n *≥**3 for all experiments. DMSO (0.1%) was used as a negative control for NOC and FSK. *P <**0.05 versus DMSO control.

Previous studies examining the activation of CFTR have shown an involvement of microtubules, and this may be tissue‐specific (Tousson et al. 1996). We recently reported that the microtubule destabilizer, nocodazole, disrupts microtubules and inhibits CFTR mediated ion transport in T84 human colonic epithelial cells (Ao et al. 2013). Thus, we explored the role of microtubules in forskolin‐induced iodide efflux in HEK‐CFTR cells. To achieve and maintain microtubule disruption, T84 cells need to be pre‐incubated at 4°C prior to exposure to 33 *μ*mol/L nocodazole while on ice (Ao et al. 2013). However, this pre‐treatment in conjunction with the loading conditions of the iodide efflux (protocol I below), were too harsh as the HEK‐CFTR cells sloughed off during the efflux measurements. Therefore, to establish a protocol for destabilizing microtubules in HEK‐CFTR cells the distribution of detergent‐soluble (monomeric) and detergent‐insoluble (microtubule) α‐tubulin was examined by immunoblot under four conditions as described in methods: I. 4°C, 30′ to destabilize microtubules followed by incubation with nocodazole on ice 30 min and then at RT, 1 h; II. Nocodazole on ice for 30 min followed by incubation at RT, 1 h; III. 4°C for 30 min followed by incubation with nocodazole at RT, 1 h; IV. Incubation with nocodazole at RT, 1 h. In all conditions, control cells were exposed to DMSO instead of nocodazole. As shown in Fig. 3A, in all conditions, treatment with nocodazole resulted in no detectable tubulin in the detergent‐insoluble tubulin fraction and increased tubulin in the detergent‐soluble fraction (Fig. 3A). These results established that in HEK‐CFTR cells, nocodazole treatment does destabilize microtubules into tubulin monomers, and that nocodazole treatment for 1 h at RT is sufficient for microtubule disruption (bottom panel in 3A). Figure 3B shows a representative quantification of the proportion of soluble tubulin compared to total tubulin (insoluble + soluble). All treatment protocols showed similar densitometric ratios, and the values of the 4C + Ice + RT treatment protocol (top panel) are shown as an example.

Using protocol IV, we next determined if microtubule disruption affected forskolin‐induced iodide efflux via CFTR. In addition to the 1 h pre‐treatment, nocodazole was present in the efflux buffer. Figure 3C shows that nocodazole had no effect on iodide efflux either in the presence or absence of forskolin. Therefore, in HEK‐CFTR cells, activation of CFTR by forskolin is independent of microtubule interaction.

**Figure 4.**
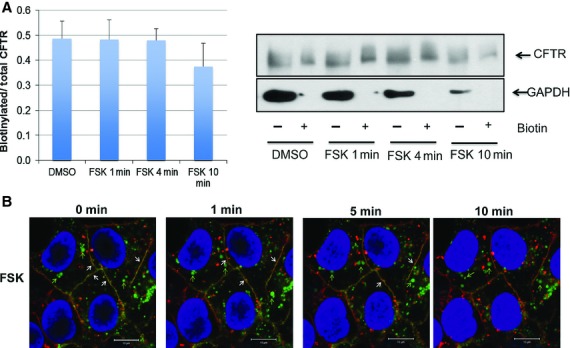
Effect of forskolin on the surface expression of CFTR. (A) Quantitation and representative blot of cell surface biotinylation of CFTR in HEK‐CFTR cells that were treated with forskolin (FSK; 10 *μ*mol/L) for 1, 4, and 10 min. GAPDH was used as a control to determine if there was any contamination of biotinylated protein in the nonbiotinylated fractions (Ab: monoclonal mouse‐anti‐CFTR‐C and anti‐GAPDH; 1:1000 and 1:2000 dilution, respectively; estimated sizes: for CFTR: 165 kDa and GAPDH: 36 kDa). (B) Effect of FSK on CFTR location in live HEK‐CFTR cells. Cells grown in 35‐mm glass‐bottom dishes were stained as described in the Methods. Images were captured before and after the treatment of FSK as time series. Snapshots at 0, 1, 4, and 10 min are shown. Green: CFTR; red: WGA; and blue: Dapi. White arrows: colocalization of CFTR and WGA; green arrows: CFTR vesicles. *n *≥**3 for all experiments.

Stimulation of ion transporters, including CFTR in some cell types, involves recruitment of intracellular vesicles to the plasma membrane (Taylor et al. 1973; Fuller et al. 1994; Tousson et al. 1996; Lotscher et al. 1997). These processes may not necessarily involve microtubules. We used two approaches, surface biotinylation and live cell imaging, to determine if forskolin causes an increase in surface expression of EGFP‐CFTR. First, HEK‐CFTR cells were treated with DMSO or forskolin for 1, 4, or 10 min and then biotinylated to capture the CFTR expressed at the plasma membrane. CFTR‐C antibody was used to screen for the proteins in the biotinylated and nonbiotinylated fractions. As shown in Fig. 4A, forskolin did not cause an increase in cell surface biotinylation at any time point and showed a slight decrease in surface expression (10 min) as determined by quantification of biotinylated CFTR (surface) to total CFTR (biotinylated + nonbiotinylated fractions). No GAPDH was detected in the biotinylated fractions, confirming the efficacy of the separation. The biotinylation results suggest that in HEK‐CFTR cells, stimulation of Cl^−^ transport does not involve an appreciable increase in CFTR recruitment to the plasma membrane, but is due to activation of CFTR already present in the membrane.

We used live cell imaging to detect the time‐dependent effects of forskolin on EGFP‐CFTR distribution. Cells were counter stained with WGA (red) for plasma membrane and DAPI (blue) for nuclei and cells were viewed by confocal microscopy over 10 min, and images captured at 25 sec intervals. As shown in Fig. 4B, the bulk of the EGFP‐CFTR is localized in intracellular vesicles with some expression on the plasma membrane. There is considerable vesicular movement, as seen in the video clips of DMSO‐treated (Video S1) and forskolin‐treated (Video S2) cells. We quantified the speed of vesicular movement at different time points (5 sec, 3, 6, and 9 min). In cells treated with forskolin the vesicular movement was significantly faster at 3 (DMSO: 0.008 ± 0.0002 *μ*m/sec; FSK: 0.016 ± 0.003 *μ*m/sec) and 6 min (DMSO: 0.006 ± 0.0002 *μ*m/sec; FSK: 0.014 ± 0.001 *μ*m/sec), and seemed to come to a halt by 9 min (DMSO: 0.005 ± 0.0003 *μ*m/sec; FSK: 0.000 *μ*m/sec). Over the time course, vesicular movement in the DMSO‐treated cells steadily decreased. We were, however, unable to detect any increase in EGFP‐CFTR in the plasma membrane as a consequence of forskolin's effects on vesicular movement.

### Inhibition of multidrug resistance protein 4 and the forskolin response in HEK‐CFTR cells

**Figure 5.**
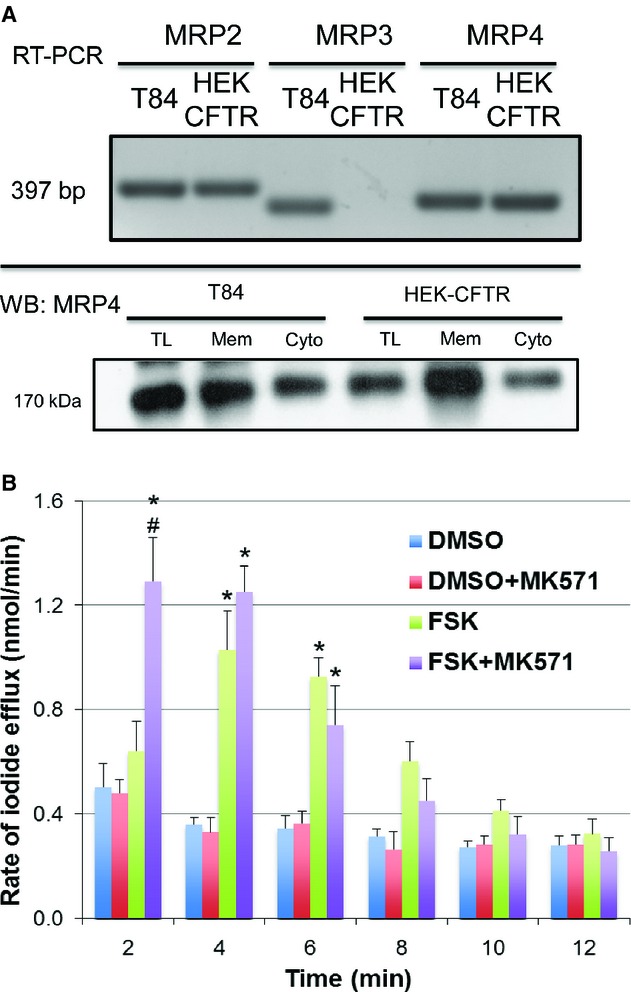
Multidrug resistance protein expression and function in HEK‐CFTR cells. (A) Upper Panel: Detection of mRNA for multidrug resistance proteins (MRPs) transcripts (MRP 2–4) by RT‐PCR; transcript size for MRPs (in bp): MRP2: 397; MRP3: 340; MRP4: 363. Lower Panel: MRP4 protein in membrane, cytosolic and total lysate fractions of HEK‐CFTR cells, determined by SDS‐PAGE, and western blotting (Ab: polyclonal goat‐anti‐MRP4; 1:750 dilution; estimated size: 170 kDa). T84 cells were used as a positive control. Blots are representative of *n *≥**3. (B) Effect of MRP inhibitor, MK571, on the rate of iodide efflux in DMSO or forskolin (FSK; 10 *μ*mol/L)‐ stimulated HEK‐CFTR cells. Cells were pretreated with the MK571 (20 *μ*mol/L) for 30 min during iodide loading, followed by stimulation with forskolin. The data represent the mean values of iodide efflux ± SEM relative to value at starting point and *n *≥**3; *P <**0.05 versus DMSO as a negative control; ^#^*P *<**0.05 versus treatment + inhibitor.

Multidrug resistance‐associated proteins (MRPs) are involved in active transport of substrates out of cells. Specifically, MRP4, a member of the ATP‐binding cassette transporter family, has been shown to act as a cAMP transporter and cause cAMP to efflux out of HT29, and T84 cells (Li et al. 2007; Xie et al. 2011). Furthermore, Li et al. (2007) demonstrated that MRP4 forms a macromolecular complex with CFTR via the scaffolding protein PDZK1 allowing for local compartmentalized regulation. We screened for MRP expression in HEK‐293 cells and found that they express the mRNA transcript for MRP2 and MRP4, but not for MRP3; they also express MRP4 protein (Fig. 5A). To determine if MRP plays a role in forskolin's activation of CFTR, we examined the effects of the MRP inhibitor MK571 (20 *μ*mol/L) on iodide efflux ± 10 *μ*mol/L forskolin. HEK‐CFTR cells were pretreated with MK571 for 30 min during iodide loading and remained in the efflux buffer during the experiment. While MK571 did not alter iodide efflux in DMSO‐treated cells, it increased the rate of iodide efflux by forskolin at 2 min when compared to forskolin alone (Fig. 5B). However, we were unable to observe any increases in total cell cAMP in response to MK571 treatment (data not shown). These results suggest that MRP4 is accentuating the forskolin effects. Whether it does this by serving as a cAMP transporter in a specific compartment remains to be determined.

### LCA does not increase iodide efflux

**Figure 6.**
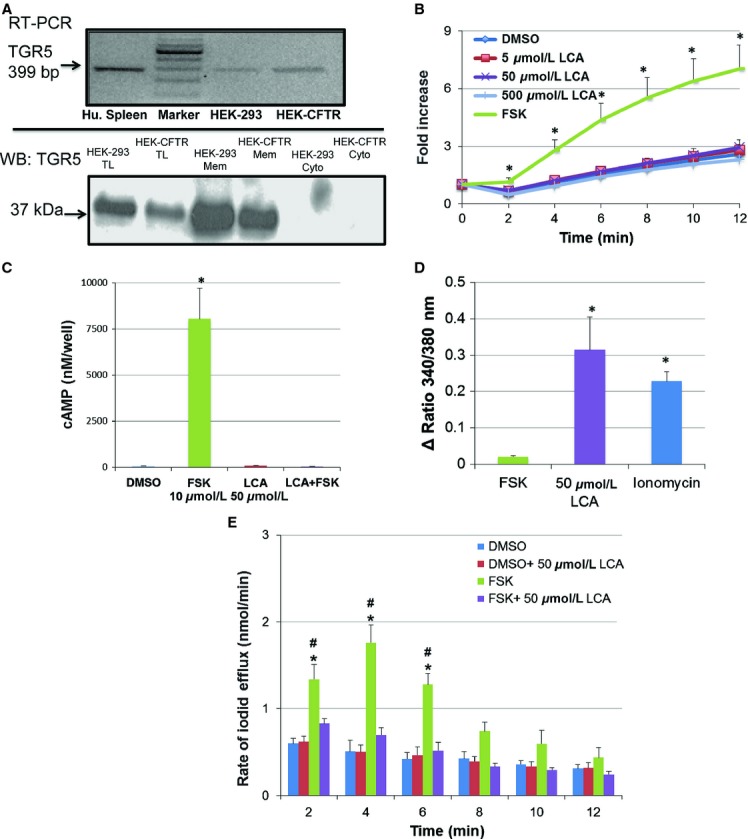
Expression and function of the bile acid G‐protein coupled receptor, TGR5, in HEK‐CFTR cells. (A) Detection of mRNA and protein for TGR5, transcript and protein by RT‐PCR and immunoblot, respectively, in HEK‐293 and HEK‐CFTR cells. Human spleen (Hu.spleen) is used as a positive control for RT‐PCRs. The cytosolic, membrane, and total lysate fractions of HEK‐293 and HEK‐CFTR were screened for the presence of TGR5 protein using a polyclonal rabbit‐anti‐TGR5 antibody (1:2500 dilution; estimated size: 37 kDa). (B) Cumulative iodide efflux was measured in the presence of the TGR5 activator, lithocholic acid (LCA; 5–500 *μ*mol/L), and forskolin (FSK; 10 *μ*mol/L) was used as positive control. The data are represented as the fold change of mean cumulative iodide efflux ± SEM relative to value at starting point in HEK‐CFTR cells; *n *≥**3. (C) HEK‐CFTR cells grown in 96‐well plates were incubated with 0.1% DMSO, 50 *μ*mol/L LCA for 15 min, or 10 *μ*mol/L FSK for 5 min, or a combination of LCA pretreatment then stimulation with FSK for [cAMP]_i_, measurements. Cyclic AMP was measured using DiscoveRx enzyme fragment complementation technology (see Methods) and is depicted as nmol/L/well. (D) HEK‐CFTR cells grown in 35‐mm glass‐bottom dishes were loaded with Fura‐2AM as described in the Methods, and the effects of 10 *μ*mol/L FSK, 50 *μ*mol/L LCA and 3 *μ*mol/L inonomycin on [Ca^2+^]_i_ were assessed as ratios (Ex_λ_: 340 and 380 nm, and Em_λ_: 505 nm). (E) Rate of iodide efflux stimulated with forskolin ± LCA (50 *μ*mol/L) in HEK‐CFTR cells. Cells were pretreated with or without LCA for 15 min during iodide loading. DMSO was used as control. LCA remained in the efflux buffer throughout the experiment and 10 *μ*mol/L forskolin (FSK) was added at *t *=**0. Data represent the mean rate of efflux (nmol/min) ± SEM relative to value at starting point. *n *=**4. *P <**0.05 versus DMSO as a negative control; ^#^*P *<**0.05 versus forskolin +LCA.

As mentioned in the introduction, a goal in establishing the HEK‐CFTR cell line was to use it as a model to examine the modulation of CFTR activity by a variety of secretagogues, including bile acids. Recently, HEK‐293 cells stably transfected with the heptahelical G protein (G*α*_s_)‐coupled membrane bile acid receptor, TGR5, were reported to respond to taurolithocholic acid with an increase in cAMP (Jensen et al. 2013). We first explored if HEK‐293 cells express endogenous TGR5 receptors for bile acids. As shown in Fig. 6A, both HEK‐293 and HEK‐CFTR cells express the transcript and protein for TGR5 and the protein is localized in the membrane fraction.

To determine if this receptor is functional, we examined the effects of a potent activator of TGR5, lithocholic acid (LCA) in HEK‐CFTR cells. At concentrations known to activate TGR5‐related functions in other cells, 5–500 *μ*mol/L LCA had no effect on iodide efflux in HEK‐293 (data not shown) or in HEK‐CFTR cells (Fig. 6B). Surprisingly, neither did exposure to 50 *μ*mol/L LCA for 15 min alter intracellular cAMP concentrations (Fig. 6C). However, LCA increased intracellular Ca^2+^ (Fig. 6D). These results suggest that although TGR5 is present in HEK‐CFTR cells, it does not functionally respond to LCA in a G*α*_s‐_dependent manner. Recent reports have suggested that while LCA does not elicit any direct effects on Cl^−^ transport, it can modulate the action of other secretagogues (Ward et al. 2013). We therefore examined the effect of LCA pretreatment on forskolin action. As shown in Fig. 6C, LCA completely inhibited the forskolin‐stimulated production of cAMP (Fig. 6C) and forskolin‐stimulated iodide efflux (Fig. 6E).

## Discussion

Epithelial Cl^−^ transport and its regulation have been characterized in a number of reductionist human cell line models derived from a variety of tissues. A composite picture is emerging with the caveat that there is considerable tissue and cell line variation in the underlying regulatory cascades. The additional caveat is the complexity in translating this information from reductionist models into what occurs in human physiological and pathophysiological states. Nevertheless, the information from cell lines is invaluable and cell lines can be readily manipulated biochemically, genetically and pharmacologically. However, some cell lines, in particular those of intestinal origin, do not lend themselves readily to genetic manipulations, such as stable transfections, while retaining polarity and function. Therefore, to better understand the molecular regulation of Cl^−^ secretion, we explored the utility of HEK‐293 cells, well established for their versatility as a model for molecular manipulations.

In a variety of secretory epithelia, CFTR is the major luminal Cl^−^ channel responsible for Cl^−^ secretion. CFTR is a linear, low conductance Cl^−^ channel, with a voltage‐independent conductance, which requires phosphorylation by cAMP‐dependent protein kinase (PKA) for activation. CFTR complexes with a number of scaffolding proteins and regulators and has been the subject of intense investigation. HEK‐293 cells are frequently used to study CFTR function and regulation in an overexpression system (Penmatsa et al. 2010). Generally, however, HEK‐293 cells are used as a tool to explore a step in a series of regulatory interactions. Thus, although Cheng et al. (2010) used HEK‐293 cells transfected with GFP‐CFTR to examine CFTR interaction with CFTR associated ligand, the accompanying transport studies were done in CFBE14o^−^, a bronchial derived cell line. However, Mondini et al. (2012) used HEK‐293 cells to examine the effect of a hypertension‐linked mutation of *α*‐adducin on CFTR surface expression and channel activity. While in these studies, the HEK‐293 cells were stably transfected with wild type or mutant *α*‐adducin, they were transiently transfected with a bicistronic vector, pIRES2‐CFTR‐EGFP, which expressed separate EGFP and CFTR proteins. CFTR activity was assessed by whole cell patch experiments. In the study by Penmatsa et al. (2010), HEK‐293 cells were used as a tool to study the physical and functional interaction between phosphodiesterase 3A and CFTR. CFTR function was assessed by two functional assays, short circuit current measurements in polarized Calu3 cells and iodide efflux measurements in HEK‐293 cells overexpressing HA‐tagged wild‐type CFTR in response to adenosine.

In the present study, we have further validated these findings by developing and characterizing a stably transfected HEK‐EGFP‐CFTR cell line. This allows us to perform consistent measurements over time. We demonstrate that the EGFP‐CFTR is functional since HEK‐CFTR but not HEK‐293 cells respond to the cAMP activator forskolin with a robust increase in iodide efflux, a measure of Cl^−^ transport. The lack of response in HEK‐293 cells is because they neither express CFTR transcript nor CFTR protein. In HEK‐CFTR cells, the protein is associated with membrane and intravesicular compartments and can be readily visualized. Our data demonstrate that the effects of forskolin are due to its action on CFTR and not on other endogenously expressed Cl^−^ channels. Minimally, HEK‐293 cells possess transcripts for two other Cl^−^ channels, ClC‐2 and the Ca^2+^‐activated Cl^−^ channel, TMEM16A. There is evidence that these channels may be involved in Cl^−^ secretion (Cuppoletti et al. 2004; Kunzelmann et al. 2012); however, in our hands, they did not respond to forskolin‐stimulation in HEK‐293 cells. It must be noted that the efficacy of the iodide efflux assay in assessing ClC‐2 or TMEM16A function is less well documented. However, studies examining the action of TMEM16A using iodide influx with a halide‐sensitive yellow fluorescent protein, established that TMEM16A can effectively transport iodide (Iosco 2012). Expression of TMEM16A in Axolotl oocytes showed halide permeabilities of iodide (3.8)> bromide (2.0) > chloride (1.0) (Schroeder et al. 2008). Finally, in HEK‐293 cells transfected with endothelin receptor subtype‐A and mouse anoctamin 1 (a.k.a. TMEM16A), the latter showed the following halide permeabilities: iodide (1.85)> bromide (1.74) > chloride (1.0)>fluoride (0.43) (Yang et al. 2008).

In HEK‐CFTR cells, forskolin increases intracellular cAMP but not Ca^2+^. Therefore, it is not surprising that the Ca^2+^ chelator BAPTA does not inhibit forskolin‐stimulated iodide efflux. It is of interest to note that in the T84 colon carcinoma cell line, forskolin causes a small but significant increase in intracellular Ca^2+^(Merlin et al. 1998; Hoque et al. 2010). The actions of forskolin in HEK‐CFTR cells are inhibited by the specific CFTR inhibitor, CFTR_inh_172 and by the PKA inhibitor H89. However, neither of these compounds completely inhibited the actions of forskolin. Variability in the ability of CFTR_inh_172 to block CFTR activity in different cell types using the iodide efflux assay has been reported (Sheppard 2012). On the other hand, Mondini et al. (2012) reported that CFTR_inh_172 could completely block the whole cell current attributable to transiently transfected CFTR in these HEK‐293 cells. The variability in efficacy of CFTR_inh_172 could either be due to differences in the source of the inhibitor, the relative insolubility of the compound in aqueous solution, or in the structure function relationship of the EGFP‐CFTR protein.

Many elegant studies have demonstrated that CFTR, via protein–protein interactions with scaffolding and regulatory proteins, exists as a macromolecular complex. This allows for fine‐tuning and spatiotemporal coupling of responses. One such coupling is between CFTR and MRP4 in HT29 colon carcinoma cells, where MRP4 functions as a cAMP transporter (Li et al. 2007). In HT‐29 cells, adenosine increases cAMP and activates CFTR, and inhibition of MRP4, accentuates this response by preventing cAMP extrusion. Our studies suggest that MRP4 may be involved in a similar manner, though its inhibition only caused a moderate increase in iodide efflux and did not increase forskolin‐stimulated cAMP production. It is possible that the discrepancy between our findings and those of Li et al. is that our assay assessed global cAMP production whereas they measured cAMP production in localized regions of the cell. Future studies using the FRET‐based EPAC‐cAMP sensor will be needed to clarify this.

Other studies demonstrate that in some, but not all, cell types, activation of CFTR includes membrane trafficking and involves microtubules interaction and remodeling. Thus, Loffing et al. (1998) showed that cAMP activation of CFTR involves increased exocytosis in intestinal but not airway epithelial cells. We recently demonstrated that in T84 cells, the stimulation of Cl^−^ transport by the bile acid chenodeoxycholate is PKA‐dependent and partially inhibited by disruption of microtubules (Ao et al. 2013). However, in HEK‐CFTR cells, despite the fact that nocodazole disrupts microtubules, it had no effect on forskolin‐stimulated Cl^−^ transport. In view of the known differences in microtubule‐dependent regulation of CFTR in intestinal versus respiratory epithelial cells, it is not surprising that HEK‐CFTR cells behave like respiratory epithelia. However, microtubule‐independent membrane trafficking can occur. Surprisingly, it appears that forskolin's activation of Cl^−^ transport in HEK‐CFTR does not alter any measurable changes in CFTR trafficking to the plasma membrane. A time course of surface biotinylation could not detect a change in trafficking in response to forskolin. Similarly, we were unable to detect increased EGFP‐CFTR in the plasma membrane in response to forskolin with live cell imaging. The lack of trafficking is not due to a paucity of CFTR‐containing membrane vesicles, since EGFP‐CFTR is prominently distributed in intracellular vesicles in HEK‐CFTR cells. However, forskolin clearly increased the rate of vesicular movement in the cell. A further characterization of the molecular basis of this vesicular movement or its biphasic regulation by forskolin is warranted. For example, it remains to be determined if the inability of this increased movement to result in increased plasma membrane expression is a consequence of HEK cell scaffolding machinery or the EGFP‐CFTR construct per se. It is conceivable that the EGFP moiety at the N‐terminus of CFTR, despite a 2 amino acid spacer, may hinder some aspects of CFTR's interaction with other protein partners, such as syntaxin, and thereby prevent trafficking.

We also examined the effect of an endogenous GPCR‐Gα_s_ ‐ cAMP‐signaling cascade on EGFP‐CFTR function. Although HEK‐CFTR cells possess the transcript and protein for the bile acid GPCR, TGR5, the TGR5 activator, lithocholic acid (LCA), failed to increase cAMP production and to stimulate iodide efflux in these cells. However, LCA caused a modest increase in [Ca^2+^]_i_. It is possible that in this cell line, the endogenous TGR5 is functionally coupled to G*α*_q,_ however, this link is less well characterized than TGR5 being linked to production of cAMP. Studies performed in the human enteroendocrine cell line NCI‐H716 showed that the synthetic TGR5 agonist INT‐777 increased calcium influx, which was reduced by TGR5 RNA interference. This study suggested that activation of TGR5 and production of cAMP was upstream of calcium influx, as inhibition of adenylyl cyclase significantly reduced the calcium influx induced by INT‐777 (Thomas et al. 2009). The lack of an effect of LCA on cAMP in HEK‐CFTR cells containing endogenous TGR5 (present study) is in contrast to the report of Jensen et al. (2013) that HEK‐293 cells stably transfected with TGR5, show increases in cAMP in response to LCA. There are perhaps two reasons for this discrepancy: first, the stably transfected cells probably have many more receptors than are present in the nontransfected cells; second, our confocal microscopy studies suggest that although present, TGR5 appears to be largely confined to intracellular vesicles (data not shown). However, while TGR5 receptor in HEK‐CFTR cells may not be “functionally‐linked” to G*α*_s_, it remains to be determined if it still plays a role in the inhibitory effects of LCA on forskolin‐stimulated iodide efflux in these cells. In other words, could LCA act via TGR5 receptor, to activate G*α*_q_, increase intracellular Ca^2+^,and thereby completely inhibit forskolin‐stimulated iodide efflux and production of cAMP? It is of interest that Ward et al. (2013) recently reported that LCA inhibits carbachol, but not forskolin‐stimulated Cl^−^ transport in rat colon. Explorations of the mechanism by which LCA is acting, the cross talk between the LCA and forskolin signaling cascades, and whether this involves spatiotemporal coupling and macromolecular complexes will be the focus of future studies.

In summary, we have characterized the HEK‐(EGFP)‐CFTR cell line as a viable model for studying the regulation of Cl^−^ transport. However, we are cognizant that this may not be entirely representative of a native epithelium. First, HEK‐293 cells are not polarized. Second, there is the issue of the cellular origin of HEK‐293 cells; some studies have revealed them to be of epithelial origin (Chan et al. 1997), and others debate their kidney nomenclature, or if they are more fibroblast‐like in nature. However, through a serendipitous series of studies, Shaw et al. (2002) have provided evidence that they are more similar to differentiating neurons in early stages. Therefore, when designing experiments using this cell line as a tool to study exogenous proteins, the endogenous signaling machinery and their possible origin should be taken into consideration.

## Acknowledgments

We thank Dr. Ravinder Gill and Ms. Tarunmeet Gujral for their advice on the biotinylation studies and Dr. R. Gill for her advice on live‐cell imaging studies. We thank Dr. Ke Ma of the Imaging Center in the Research Resources Center, UIC for her help with the confocal microscopy studies.

## Conflict of Interest

None declared.

## Supplementary Material

Supplementary Video S1

Supplementary Video S2
